# Right Ventricle Remodeling Metabolic Signature in Experimental Pulmonary Hypertension Models of Chronic Hypoxia and Monocrotaline Exposure

**DOI:** 10.3390/cells10061559

**Published:** 2021-06-21

**Authors:** Thaïs Hautbergue, Fabrice Antigny, Angèle Boët, François Haddad, Bastien Masson, Mélanie Lambert, Amélie Delaporte, Jean-Baptiste Menager, Laurent Savale, Jérôme Le Pavec, Elie Fadel, Marc Humbert, Christophe Junot, François Fenaille, Benoit Colsch, Olaf Mercier

**Affiliations:** 1 Département Médicaments et Technologies pour la Santé (MTS), Université Paris-Saclay, CEA, INRAE, SPI, MetaboHUB, 91191 Gif-sur-Yvette, France; thautbergue@gmail.com (T.H.); Christophe.JUNOT@cea.fr (C.J.); Francois.FENAILLE@cea.fr (F.F.); Benoit.COLSCH@cea.fr (B.C.); 2 Faculté de Médecine, Université Paris-Saclay, 91191 Gif-sur-Yvette, France; antignyfabrice@gmail.com (F.A.); a.boet@ghpsj.fr (A.B.); bastienmasson999@gmail.com (B.M.); melanie.lambert91@hotmail.fr (M.L.); jb.menager@ghpsj.fr (J.-B.M.); laurent.savale@gmail.com (L.S.); lepavec@gmail.com (J.L.P.); fadelelie5@gmail.com (E.F.); marc.humbert@aphp.fr (M.H.); 3 INSERM UMR_S 999 Hypertension Pulmonaire: Physiopathologie et Nouvelles Thérapies, Hôpital Marie Lannelongue, 92350 Le Plessis-Robinson, France; 4 Service de Réanimation des Cardiopathies Congénitales, Hôpital Marie Lannelongue, Groupe Hospitalier Paris Saint Joseph, 92350 Le Plessis-Robinson, France; 5 Cardiovascular Medicine, Stanford Hospital, Stanford University, Stanford, CA 94305, USA; fhaddad@stanford.edu; 6 Service d’Anesthésie, Hôpital Marie Lannelongue, Groupe Hospitalier Paris Saint Joseph, 92350 Le Plessis-Robinson, France; amelie-delaporte@hotmail.com; 7 Service de Chirurgie Thoracique, Vasculaire et Transplantation Cardio-Pulmonaire, Hôpital Marie Lannelongue, Groupe Hospitalier Paris Saint Joseph, 92350 Le Plessis-Robinson, France; 8 Assistance Publique—Hôpitaux de Paris (AP-HP), Service de Pneumologie et Soins Intensifs Respiratoires, Centre de Référence de l’Hypertension Pulmonaire, Hôpital Bicêtre, 94270 Le Kremlin-Bicêtre, France

**Keywords:** RV dysfunction, MCT, chronic-hypoxia, arginine, tryptophan, purine

## Abstract

Introduction: Over time and despite optimal medical management of patients with pulmonary hypertension (PH), the right ventricle (RV) function deteriorates from an adaptive to maladaptive phenotype, leading to RV failure (RVF). Although RV function is well recognized as a prognostic factor of PH, no predictive factor of RVF episodes has been elucidated so far. We hypothesized that determining RV metabolic alterations could help to understand the mechanism link to the deterioration of RV function as well as help to identify new biomarkers of RV failure. Methods: In the current study, we aimed to characterize the metabolic reprogramming associated with the RV remodeling phenotype during experimental PH induced by chronic-hypoxia-(CH) exposure or monocrotaline-(MCT) exposure in rats. Three weeks after PH initiation, we hemodynamically characterized PH (echocardiography and RV catheterization), and then we used an untargeted metabolomics approach based on liquid chromatography coupled to high-resolution mass spectrometry to analyze RV and LV tissues in addition to plasma samples from MCT-PH and CH-PH rat models. Results: CH exposure induced adaptive RV phenotype as opposed to MCT exposure which induced maladaptive RV phenotype. We found that predominant alterations of arginine, pyrimidine, purine, and tryptophan metabolic pathways were detected on the heart (LV+RV) and plasma samples regardless of the PH model. Acetylspermidine, putrescine, guanidinoacetate RV biopsy levels, and cytosine, deoxycytidine, deoxyuridine, and plasmatic thymidine levels were correlated to RV function in the CH-PH model. It was less likely correlated in the MCT model. These pathways are well described to regulate cell proliferation, cell hypertrophy, and cardioprotection. These findings open novel research perspectives to find biomarkers for early detection of RV failure in PH.

## 1. Introduction

Regardless of the type of pulmonary hypertension (PH), the right ventricle (RV) initially adapts to pressure overload and is able to maintain a normal cardiac output [1,2,3]. Over time and despite optimal medical management, RV function deteriorates from adaptive to maladaptive phenotype, leading to right ventricle failure (RVF). Although RV function being a well-recognized prognostic factor of PH [4,5], no predictive factor of RVF has been elucidated so far [6]. However, several studies demonstrated that the adaptive to maladaptive phenotype shift involved defective glycolysis, RV capillary rarefaction, RV fibrosis, and RV inflammation [7,8,9]. We hypothesized that a global RV metabolic alterations study might help find new biomarkers for the early identification of PH-associated RV failure. The metabolome is the set of metabolites (small molecules, <1000 Da) found in biological media as opposed to metabolomics which deals with the large-scale detection of metabolites in biological samples. High-resolution mass spectrometry (HRMS) is preferentially used for metabolomics studies. It seems adapted to biomarkers screening as it has proved more efficient than low-resolution instruments in detecting and identifying metabolites [10]. Publications on metabolic alterations of PH patients’ hearts remain scarce. In 2016, Rhodes et al. [11] investigated metabolic alterations in PH patients’ plasma with mass spectrometry showing alterations of energy metabolism, tryptophan, and polyamine metabolic pathways. However, none of these alterations have been correlated to RV function or remodeling of the phenotype. Similarly, other studies limited their investigation to the plasmatic signature of PH patients considering the difficulty of collecting myocardial biopsies [12,13]. Of interest, studies on animal models focus on metabolic alterations in plasma without myocardial correlation analyses. Zheng et al. [14] analyzed plasma samples from monocrotaline (MCT)-PH rats and showed altered arginine metabolism. Zhao et al. analyzed plasma samples of both MCT-PH and Chronic-hypoxia (CH)-PH rat and showed that methionine metabolism and urea cycle metabolism were the most significant pathway involved in the pathogenesis of PH induced by MCT or CH-exposure [15].

The current study aimed at characterizing metabolic reprogramming associated with RV remodeling the phenotype during PH in order to give new insights into physiopathological processes involved in maladaptive RV remodeling and to highlight putative biomarkers for the early detection of RV failure. For that purpose, we analyzed RV and LV tissues in addition to plasma samples from MCT-PH and CH-PH rat models. We used an untargeted metabolomic approach based on liquid chromatography coupled to HRMS [16]. Plasma and myocardial metabolic signatures were compared with the goal of new RV-specific biomarker discovery in PH.

## 2. Material and Methods

### 2.1. Animals

The animal facility is licensed by the French Ministry of Agriculture (agreement N° C92-019-01). This study was approved by the Committee on the Ethics of Animal Experiments (CEEA26 CAP Sud). Animal experiments were performed according to the guidelines from Directive 2010/63/EU on 22 September 2010 of the European Parliament on the protection of animals used for scientific purposes and complied with the French institution’s guidelines for animal care and handling. Male Wistar rats (4 weeks old) were used in four experimental protocols: (1) PH was induced by a single MCT injection (60 mg/kg, subcutaneous) in 14 rats. MCT was dissolved in 1 N HCl and neutralized with 1 N NaOH. (2) 14 Control rats received the same volume of saline solution (0.9% NaCl) (designated here as “vehicle rats”). (3) For CH-exposure, 10 Wistar rats were placed for 3 weeks in hypoxia (10% O_2_) and normobaric conditions. (4) Ten Normoxia Wistar rats were placed in the same room for 3 weeks as controls.

### 2.2. Echocardiographic Measurement

Evaluation by trans-thoracic echocardiography (TTE) was performed with a digital ultrasound system (Vivid E9, GE Healthcare, Buc, France) by using a high-frequency phased array transducer (12 S-D 4-12MHz, GE Healthcare, Buc, France). The echocardiographic evaluation procedure was performed under general anesthesia and spontaneous breathing with an Isoflurane Rodent Anesthesia System (Minerve, Esternay, France) (maintenance isoflurane 2% at room air). Rats were shaved, and body temperature was controlled during experiments. All analyses were performed in a blinded manner: rats’ experimental conditions were unknown by the operator during TTE examination and data interpretation. In the parasternal short-axis view, we measured pulmonary artery acceleration time (PAAT; ms), pulmonary artery velocity time integral (VTI-PA), RV ejection time (RVET; ms), cycle length (ms) as previously described [17]. In the 4-cavity view, performed subcostally, we measured ascending aorta velocity time integral (VTI-Aorta (VTI-Ao)) reflecting LV cardiac output [18,19], VTI-Ao value reproducible in heart failure [20,21], Tricuspid annular plane systolic excursion (TAPSE; mm), RV and LV thickness (mm). RV and LV fractional shortening (RV FS and LV FS%) corresponded to the percent change in LV and RV cavity diameters, respectively. LV FS = ((LV EDd-LV EDs/LV EDd)*100 or RV FS = (RV EDd-RV EDs/RV EDd)*100, as previously described [17]. Echocardiographic measurements were illustrated in Supplementary Figure S1.

### 2.3. Right Heart Catheterization and Tissues Harvest

Rats were placed under general anesthesia and spontaneous breathing with an isoflurane Rodent Anesthesia System (Minerve Esternay, France) (maintenance: isoflurane 2% at room air). Hemodynamic measurements were performed, such as cardiac output (CO; mL/min), heart rate (HR; beat/min), and Min and Max dPdt (MindPdt and MaxdPdt, mmHg/s). Recording of the right ventricular pressure (RVP) allowed the measurement of the right ventricular systolic pressure (RVS.P; mmHg). Pulmonary vascular resistance (PVR) was assessed by the ratio of RVS.P and cardiac output. Hemodynamic parameters were blindly analyzed in unventilated anesthetized rats using LabChart software. LabChart allowed us to calculate several ventricular wave form parameters, including End diastolic pressure (EDP), Contractility index which corresponds to Max dP/dt divided by the RV pressure (RVP), at the time of Max dP/dt. Four rats from the MCT sample sets died before catheterization. Therefore, their RVS.P, HR, Max and MindPdt, CO, and PVR could not be assessed. After the catheterization, plasma, RV and LV were collected and stocked at −80 °C before metabolomic analyses. Fulton’s index was calculated before sample congelation: the ratio of RV weight to LV plus septal (S) weight (RV/LV+S). 

### 2.4. Pulmonary Vessel Remodeling Analysis

The lungs were fixed in 4% paraformaldehyde, embedded in paraffin, and serially sectioned (5 μm). The histopathological evaluation of the lungs was performed by a pulmonary pathologist who was blinded to the genotypes and treatment group assignments of the rats. The morphometric analyses were performed on sections stained with hematein–eosin–safran (HES). The lung samples from all the conditions were analyzed by conventional light microscopy using quantitative semi-automated software (NIS-BR; Nikon, France). Vascular remodeling was assessed in all the pulmonary vessels larger than 50 μm and less than 100 μm that were identified in 20 randomly selected microscopic fields. The wall thickness was calculated according to the following equation: ((External diameter − Internal diameter)/(External diameter)) × 100.

### 2.5. RV Fibrosis Assessment

RV were fixed in 4% paraformaldehyde, paraffin-embedded, and serially sectioned (5 μm). The sections were stained with trichrome Masson (#MST-100T Biognost) to evaluate fibrosis as a percentage of the total tissue area % of fibrosis was measured using Image J software (Bethesda, Rockville, MD, USA).

### 2.6. Sample Preparation for LC-HRMS Analysis

Please find more details about methods in Supplementary Material—From plasma: Metabolites were extracted from 50 µL of plasma as previously described [16]. A quality control (QC) sample was obtained by pooling 20 µL of each sample. Briefly, 50 μL of plasma were treated with 200 μL of cold methanol containing internal standards and left on ice for 90 min to allow protein precipitation. Supernatants were then collected and dried. Prior to LC-HRMS analysis, dried extracts were dissolved in 150 µL of H2O/Acetonitrile (95:5, *v/v*), containing 0.1% formic acid + external standards or 10 mM ammonium carbonate pH 10.5 + external standards/Acetonitrile (40:60, *v/v*) for metabolite analysis using C18 and ZIC-pHILIC columns, respectively. QC samples were injected every 10 samples in order to evaluate the signal variations of any metabolite. *From heart tissues*: Metabolites were extracted from 30 mg of ground ventricle resuspended in 170 µL of ultrapure water by sonication 5 times for 10 s. At this step, the total protein concentration was determined. A volume of 350 µL of methanol containing internal standards was added to the tissue lysate, which was then left on ice for 90 min for protein precipitation. Cell debris were then removed by centrifugation, and supernatants were split into two equal aliquots for C18 and HILIC analyses. The resulting aliquots were then dried and stored at −80 °C until analysis. Prior to LC-MS analysis, dried extracts were resuspended to reach a fixed protein concentration (equivalent to 20 mg/mL) using variable volumes of H2O/Acetonitrile (95:5, *v/v*), containing 0.1% formic acid + external standards or 10 mM ammonium carbonate pH 10.5 + external standards/Acetonitrile (40:60, *v/v*) for metabolite analysis using C18 and ZIC-pHILIC columns, respectively. A quality control (QC) sample was obtained by pooling 20 µL of each sample preparation.

### 2.7. LC-HRMS Conditions

Ten microliters of extracts were analyzed by LC-MS using an Ultimate 3000 chromatographic system (ThermoFisher Scientific, Courtaboeuf, 91140 France) coupled to an Exactive mass spectrometer (ThermoFisher Scientific Courtaboeuf, 91140 France) fitted with an electrospray source and operating in the positive and negative ion modes for metabolite separations on C18 (C18(+) conditions) and ZIC-pHILIC (HILIC(-) conditions) columns, respectively. Ions were detected from *m/z* 85 to 1000 [16]. Ultra-high-performance LC (UHPLC) separation was performed on a Hypersil GOLD C18 (1.9 μm, 2.1 mm × 150 mm) column at 30 °C (ThermoFisher Scientific, les Ulis, France), using a gradient program of 0.1% formic acid in water (phase A) and 0.1% formic acid in acetonitrile (phase B). High-performance LC (HPLC) separation was performed on a Sequant ZICpHILIC column (5 μm, 2.1 × 150 mm) at 15 °C (Merck, Darmstadt, Germany), using a gradient program of an aqueous buffer of 10 mM ammonium carbonate pH 10.5 (phase A) and acetonitrile (phase B). After LC-HRMS analysis, QC samples were re-injected for performing additional higher-energy collisional dissociation (HCD) tandem mass spectrometry experiments (MS/MS) in both negative and positive ionization modes, with the instrument set in the targeted acquisition mode, using inclusion lists. HCD mass spectra were inspected manually to confirm annotations of detected metabolites.

### 2.8. Data Processing and Statistical Analysis

Data processing workflow and statistical analyses were performed on the open web-based platform workfow4metabolomics, a collaborative research infrastructure for computational metabolomics (W4M: https://workflow4metabolomics.usegalaxy.fr [22], version 4.0.0, accessed on 8 July 2020). Automatic peak detection and integration were performed using the matched filter algorithm in the W4M pre-processing package (including XCMS software). XCMS features were thereafter filtered according to the following criteria: (i) a correlation between dilution factors of QC samples and areas of chromatographic peaks superior to 70%, (ii) repeatability (the coefficient of variations obtained for chromatographic peak areas of QC samples should be below 30%), and (iii) ratio of chromatographic peak area of biological to blank samples above a value of 3. Features were also annotated by matching their accurate measured masses and chromatographic retention times with our spectral database obtained from ~1000 pure, authentic standards [16]. In addition, using the present untargeted metabolomics approach, some isomeric metabolites cannot be resolved. Thus, some chromatographic peaks could correspond to more than 1 metabolite. This is typically the case for glucose, galactose, and other hexose isomers or also for pentose phosphates. MS/MS patterns of metabolites that are particularly mentioned in our work were compared with MS/MS patterns of authentic standards to validate their identification, as proposed by the Metabolomics Standards Initiative [23]. Statistical analyses were performed with W4M. Normal distribution of was evaluated with a Shapiro–Wilk normality test. Echocardiography and catheterization parameters were considered as discriminant when *p*-value < 0.05 with a *t*-test or Mann–Whitney test, applied depending on normality of measures distribution. Metabolites were considered as discriminant when showing both *p*-value < 0.05 with Mann–Whitney statistical test and VIP > 1 in multivariate analysis (OPLS-DA).

## 3. Results

### 3.1. RV Remodeling after CH and MCT-Exposure in Rats

**Figure 1:**
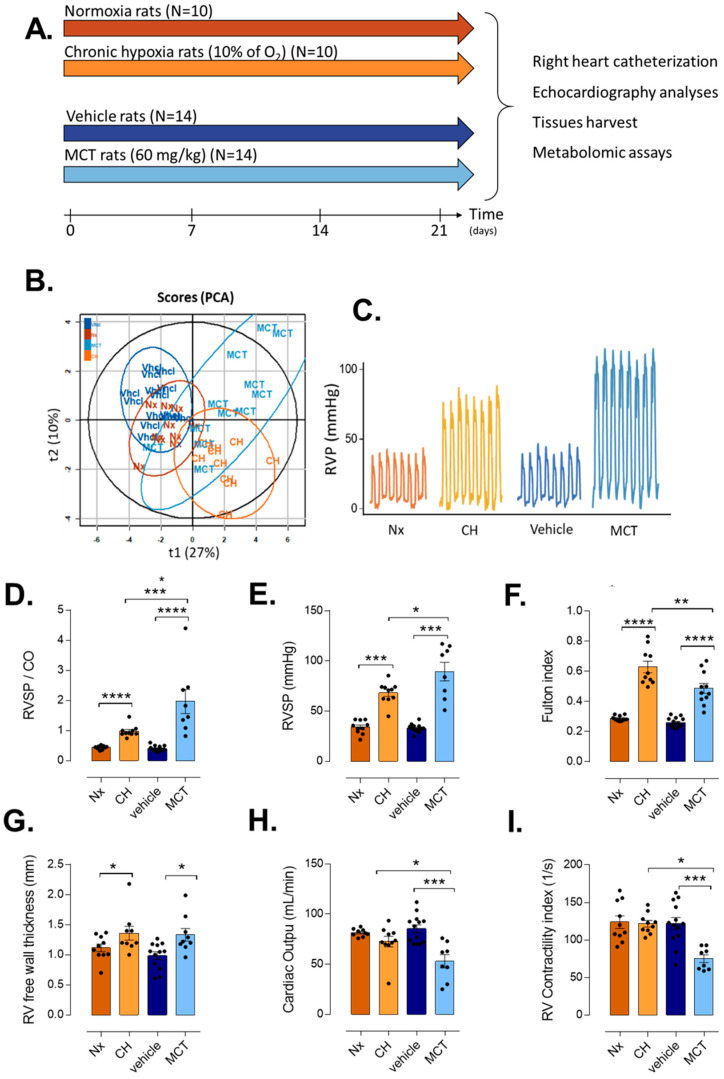
Characterization of pulmonary hypertension by echocardiography and right heart catheterization of CH-PH and MCT-PH rats and their respective controls. (**A**). Experimental protocol. Preparation and analysis of CH-PH rats, MCT-PH rats, Normoxia rats (as control for CH-PH rats), and Vehicle rats (as controls for MCT-PH). (**B**). Principal Component Analysis (PCA) projection from all echocardiography and catheterization parameters. (**C**). Representation of the record of right ventricular pressure (RVP) expressed in mmHg. (**D**–**I**). Bar graph of top-six altered parameters in normoxia (Nx), chronic hypoxia (CH), vehicle and monocrotaline (MCT) rats: (**D**). Right ventricular systolic pressure (RVS.P). (**E**). Pulmonary vascular resistance (PVR, evaluated by RVS.P/Cardiac output ratio). (**F**). RV hypertrophy by measuring the Fulton index. (**G**). RV free wall thickness index. (**H**). Cardiac output. (**I**). RV contractility index. t-tests were used after verification of normal distribution of values (Shapiro–Wilk normality test). *p*-values are shown in Supplementary Table S1. Significance: * *p ≤* 0.05; ** *p ≤* 0.01; *** *p ≤* 0.001, **** *p* ≤ 0.0001.

CH and MCT-induced PH models were compared with their respective controls (normoxia and vehicle) (Figure 1A). Principal component analysis (PCA) based on echocardiographic and right heart catheterization parameters showed similarities between normoxia and vehicle rats compared with both CH- and MCT-PH rats. Of interest, PCA showed strong differences between CH and MCT-PH rats (Figure 1B). However, both CH- and MCT-PH rats developed severe PH, while normoxia and vehicle rats showed normal pulmonary pressure. This was highlighted by the strong increase in RV systolic pressure (RVS.P) (Figure 1C,E), pulmonary vascular resistance (Figure 1D), end-diastolic pressure (EDP) (Table 1) as well as by the decrease in the pulmonary artery acceleration time (PAAT) in CH-PH and MCT-PH rats compared to their respective controls (Table 1). As a consequence, CH- and MCT-exposed rats developed RV hypertrophy as indicated by the increased Fulton’s index and RV free wall thickness (Figure 1F,G). Interestingly, CH exposure induced an adaptive RV phenotype as opposed to MCT exposure which induced a maladaptive RV phenotype. Indeed, in the CH-PH model, the RV contractility was preserved as indicated by RV contractility index values (121.7 ± 13.63 vs. 75.25 ± 14.7 for CH and MCT-PH rats, respectively) (Table 1 and Figure 1H,I) and RV hypertrophy (calculated by Fulton’s index as the weight ratio of RV and (LV + septum)) (0.63 ± 0.12 vs. 0.49 ± 0.11) was more pronounced than in MCT-PH rats (Table 1 and Figure 1F). On the opposite, RV was dysfunctional in MCT-PH rats. As a matter of fact, the elevation of RVS.P and PVR was more pronounced in MCT-PH (89.46 ± 26.02) rat group than in CH-PH rat group (68.26 ± 10.83), suggesting that PH was more severe in MCT-PH rats compared to CH-PH rats (Table 1). Moreover, cardiac output (CO) was more markedly decreased in MCT-PH-rats (Figure 1H), which could be a consequence of compromised LV filling or associated with RV dysfunction in MCT-PH rats. To support this hypothesis, we found that the RV contractility index (Figure 1I) was reduced in MCT-PH compared to CH-PH rats and control animals. We also found that the tricuspid annular plane systolic excursion (TAPSE) was decreased in MCT-PH rats and not decreased in CH- vs. normoxia rats (Table 1). No significant changes were observed in RVET between CH and MCT rats. LV echocardiography analysis revealed that LV fractional shortening (LV FS) was similar in CH-PH and MCT-PH compared to their respective controls, while the Aorta velocity integral time (VTI-Ao) was reduced in MCT-PH rats compared to vehicle-exposed rats (Table 1).

**Figure 2:**
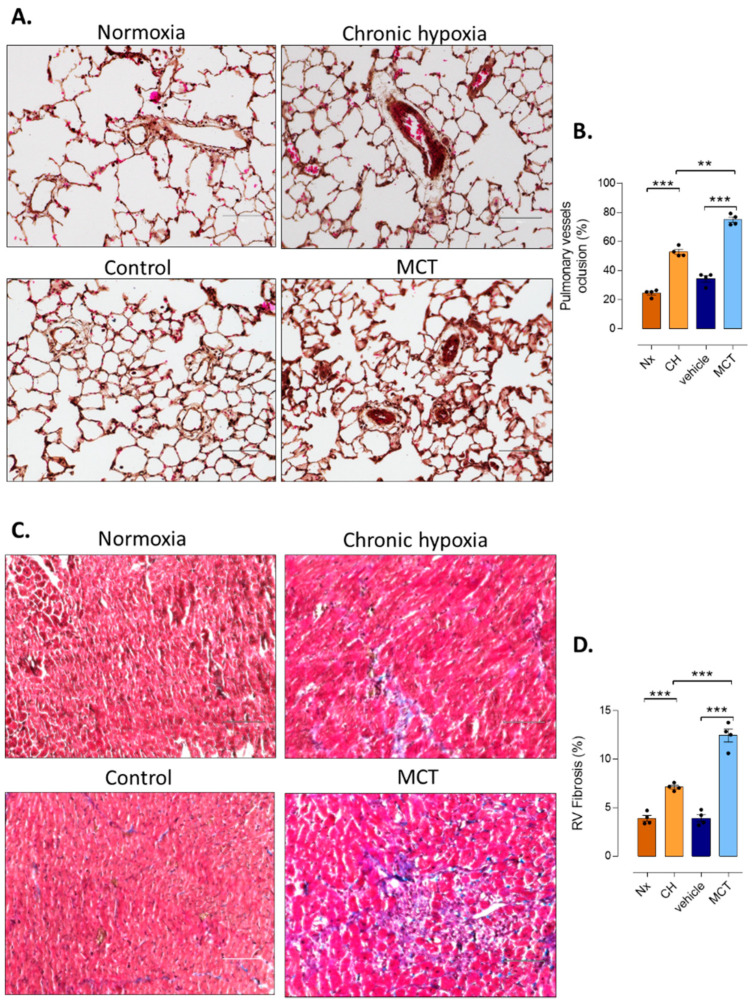
Characterization of pulmonary vascular remodeling and RV fibrosis in CH-PH and MCT-PH rats and their respective controls. (**A**). Representative hematein–eosin–safran (HES) staining of the paraffin-embedded lung sections from the control, MCT, Vehicle, Chronic hypoxia (CH), and normoxia (Nx) groups. Scale bar = 100 μm. (**B**). Pulponary vessel occlusion (%) in the control, the control, MCT, Vehicle, CH and Nx groups (n = 4 different rats per condition). (**C**). Interstitial fibrosis was identified with trichrome Masson staining in the RV compartments of control, the control, MCT, Vehicle, CH, and Nx groups. Scale bar = 100 μm (**D**). Quantification of the percentage of fibrosis in RV tissues from control and MCT-exposed rats (n = 20 images per rat from 4 rats). *t*-tests were used after verification of normal distribution of values (Shapiro–Wilk normality test). Significance: ** *p ≤* 0.01; *** *p ≤* 0.001.

In addition to hemodynamic characterization of PH, we also analyzed pulmonary vascular remodeling in MCT-PH and CH-PH rats using hematoxylin and eosin staining of paraffin-embedded lung section. We found a significant increase in the vascular wall thickness in MCT-PH rats and in CH-PH rats compared with their respective controls (Figure 2A,B). Importantly, we measured that the vascular wall thickness was more important in lung from MCT-PH rats compared with lung from CH-PH rats (Figure 2A,B).

Three weeks after PH induction by MCT or CH exposure, we also measured the presence of RV fibrosis using trichrome Masson staining (Figure 2). We found a strong increase in RV fibrosis in CH-exposed rats and MCT-PH rats (Figure 2C,D), and the RV fibrosis was more pronounced in RV from MCT-PH compared with RV from CH-PH rats (Figure 2C,D).

### 3.2. PH-Induced Cardiac Remodeling Associated with Alteration of Four Metabolic Pathways

KEGG database [24] was consulted to determine involved metabolic pathways (Supplementary Table S2). Arginine, purine, pyrimidine, and tryptophan metabolic pathways were altered in both models (Table 2). The arginine pathway was the most altered pathway with 18 altered metabolites in CH-PH rats (12 in plasma, 14 in RV and LV tissues) and 12 altered metabolites in MCT rats (5 in plasma, 11 in heart tissues). This pathway showed the strongest alterations with seven metabolites with fold changes greater than 1.8 in heart tissues and significant statistics with *p*-values < 0.001 for half of the significant results. Pyrimidine pathway was the second most altered pathway with 11 altered metabolites in CH-PH rats (10 in plasma and 8 in heart tissues) and 12 altered metabolites in MCT rats (7 in plasma and 10 in heart tissues). An overall increase in metabolites from the pyrimidine pathway was observed in both MCT and CH-PH rats and systematically detected in RV of both CH-PH rats and MCT-PH rats (unless two metabolites were specifically altered in plasma). As opposed to the arginine and pyrimidine pathways that were globally altered in both heart and plasma, alterations of metabolites from purine metabolism were mostly found in heart tissues. Over the 10 metabolites altered in the CH-PH model, 9 were altered in heart tissues versus 3 in plasma. Similarly, from the eight metabolites altered in MCT rats, all were altered in the heart but only two in plasma. Alteration of tryptophan metabolism followed another different pattern since half of tryptophan metabolism showed a specific plasmatic alteration. The tryptophan pathway was represented by six altered metabolites in CH-PH rats (five in plasma and four in heart tissues) and eight alterations in MCT rats (six in plasma and five in heart tissues). The results from the tryptophan pathway were less significant and difficult to interpret due to a mismatch between MCT-PH and CH-PH rats. 

### 3.3. Five Metabolites as PH-Induced Cardiac Remodeling Metabolic Signature

**Figure 3:**
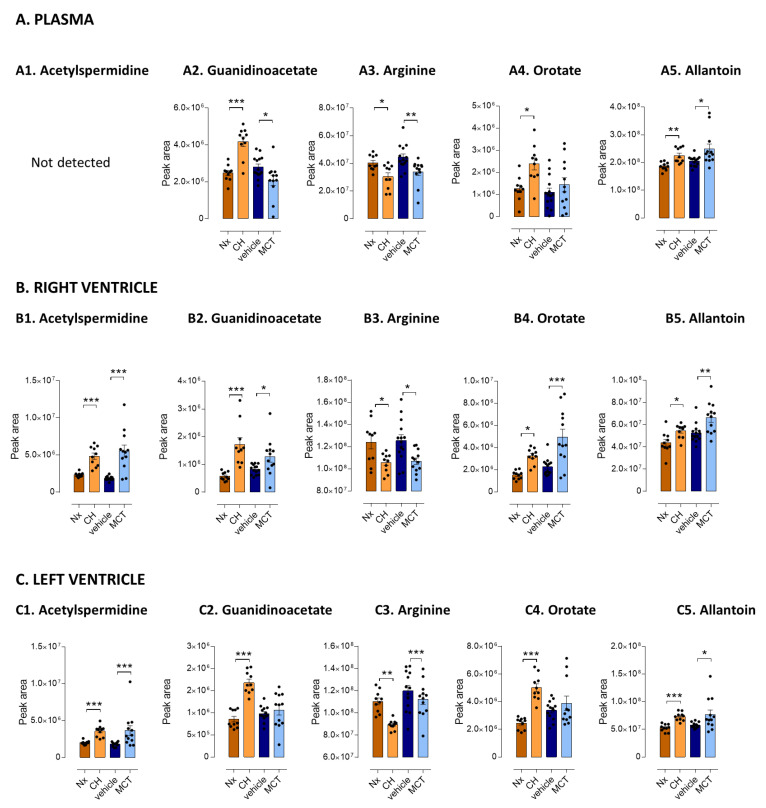
Top5 altered metabolites in PH rat models. Bar graph of metabolites discriminating chronic-hypoxia rats (CH) models from normoxia rats (Nx) and monocrotaline (MCT) rats from vehicle rats (Vhcl) (Mann–Whitney test). (**A**). In plasma. (**B**). In right ventricle. (**C**). In left ventricle. Significance versus the respective control group (Mann–Whitney test): * *p ≤* 0.05. ** *p ≤* 0.01. *** *p ≤* 0.001. Reader’s note: due to specific matrix effects, intensities in plasma and heart tissues are not comparable.

Alteration of polyamines (i.e., spermidine, acetyl-spermidine, and putrescine) was only observed in LV and RV tissues with *p*-values < 0.01, and fold changes were among the most important FC observed in the current study (Table 2). Particularly, acetyl-spermidine was found to be elevated more than twice in the heart of PH-models (Figure 3(B1,C1)). In addition, arginine and guanidinoacetate were altered in plasma and heart (LV and RV from both CH and MCT rats). Guanidinoacetate was the most altered metabolite of the arginine metabolic pathway with FC > 3 and *p* < 0.0001 in CH-PH RV compared to normoxia rats (Table 3 and Figure 3(A2,B2,C2)). Arginine decreased in both plasma and LV and RV tissues (Figure 3A3,B3,C3), in accordance with the broad literature about arginine in PH and RV failure [14,25,26,27,28,29]. Orotate was the most impacted metabolite from the pyrimidine metabolic pathway, with fold changes >2 in RV of both MCT and CH models and with highly significant *p*-values in CH-PH heart and plasma (*p* < 0.0001) (Table 2 and Figure 3(A4,B4,C4)). Concerning the purine metabolic pathway, allantoin was the only metabolite altered in plasma and LV and RV tissues of both CH-PH and MCT-PH rats (Figure 3(A5,B5,C5)). Furthermore, xanthosine, xanthine, guanosine, and inosine were specifically altered in RV of both CH-PH and MCT-PH models.

### 3.4. Focus on Arginine Bioavailability and Enzymatic Activities

Arginine bioavailability was estimated by the arginine-to-ornithine ratio (which reflects arginase activity) and arginine-over-guanidinoacetate ratio (which reflects arginine:glycine amidinotransferase activity). A significant decrease in the arginine-over-ornithine ratio illustrated a stimulation of arginase in plasma and RV of both CH-PH and MCT-PH rat models (Supplementary Figure S2). Similarly, arginine-over-guanidinoacetate ratios were reduced in plasma, RV and LV of CH-PH rat, reflecting an increase in arginine:glycine amidinotransferase activity. These ratios evaluation underlined a double source of arginine consumption, reducing arginine bioavailability.

### 3.5. Correlation between Metabolic Signature and RV Function

(arg pathway) Levels of acetylspermidine and putrescine in heart tissues were correlated to RVS.P, Fulton’s index, PVR, and PAAT in both models but particularly in CH-PH rats with overall absolute values of correlation coefficients superior to 0.7 (Table 3). Guanidinoacetate correlated with 11 echocardiography and hemodynamic parameters. (pyruvate pathway) Moreover, orotate RV levels were highly correlated with clinical parameters, since they showed R > 0.8 and *p* < 0.0001 in each organ for PVR correlation and R > 0.8 and *p* < 0.00001 in RV for RVS.P, RV weight, and Fulton’s index correlations in CH-PH rats (Table 3). Cytosine, deoxycytidine, deoxyuridine, and plasmatic thymidine levels were also highly correlated with RVS.P, RV weight, and Fulton’s index. (purine pathway) Allantoin levels were the most correlated to RVS.P, RV weight, Fulton’s index, and PVR in CH-PH rats (Table 3). Xanthosine, xanthine, guanosine, and inosine levels were not correlated with echocardiography and catheterization parameters. 

## 4. Discussion

To the best of our knowledge, this is the first time that metabolic disorders have been studied from heart tissues in PH animal models. We analyzed plasma and heart tissues with an untargeted metabolomic approach to detect and compare variations of more than 280 metabolites in two different rat models (MCT-PH and CH-PH). Metabolomic analysis showed that four main metabolic pathways (arginine, pyrimidine, purine, and tryptophan) were altered in both models. Among these metabolic pathways, many metabolite levels from arginine, pyrimidine, tryptophan, and purine pathways were highly correlated with cardiac morphometric, RV, and closed-chest heart catheterization parameters in the adaptive phenotype of CH-PH models, suggesting their involvement in PH-induced cardiac remodeling and their potential role as specific biomarkers for RV in PH. 

RV remodeling and dysfunction in MCT-PH rats have been often associated with maladaptive RV (reduction in exercise capacity, reduction in RV function, capillary rarefaction, RV inflammation). In CH-PH rats, the pulmonary arterial medial hypertrophy leads to elevated RVS.P and RV hypertrophy without RV failure [30]. In the present study, we confirmed that MCT exposure induces severe PH in rats with maladaptive RV while rats exposed to CH develop severe PH with adaptive RV. Therefore, we confirmed from plasma and heart biopsy samples that MCT and CH-rats are complementary models as suggested by Zhao et al. [15].

### 4.1. Arginine and Tryptophan Metabolic Pathways, a Duel for the Control of Pulmonary Arterial Tone in PH Plasma

**Figure 4:**
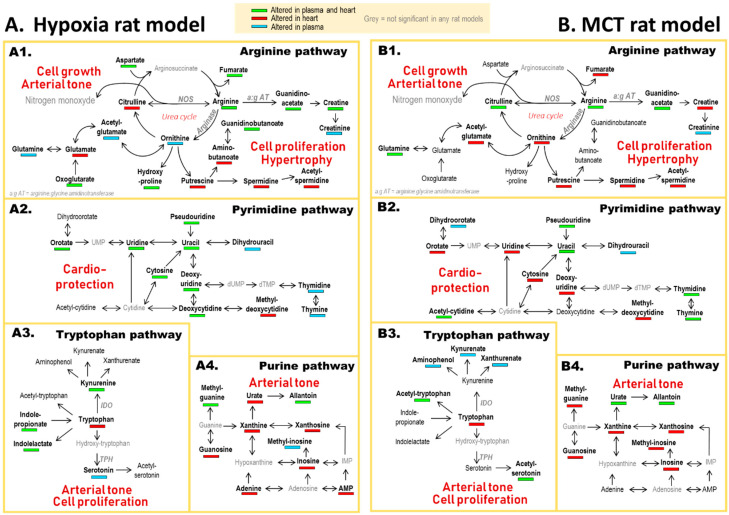
Metabolomic pathways mainly altered on CH-PH and MCT-PH rats. IDO: Indoleamine 2,3-Dioxygenase. TPH: Tryptophan hydroxylase. NOS: Nitric oxide synthase. Main impacted pathways in: (**A**) hypoxia rat model (A1: Arginine metabolic pathway, A2: Pyrimidine metabolic pathway, A3: Tryptophan metabolic pathway and A4: Purine metabolic pathway) and (**B**) MCT rat model (B1: Arginine metabolic pathway, B2: Pyrimidine metabolic pathway, B3: Tryptophan metabolic pathway and B4: Purine metabolic pathway). Green color: metabolites altered in both heart and plasma samples. Red color: metabolites altered only in heart samples. Blue color: metabolites altered only in plasma samples.

Arginine metabolism was the most impacted pathway in accordance with previous studies that described such alteration in plasma [12,15] and lungs of MCT-PH rats [25]. In the urea cycle, arginine can be metabolized competitively by nitrite oxide synthase (NOS) to produce nitric oxide (NO) or by arginase to synthesize ornithine and then polyamines (Figure 4(A1,B1)). This competitive metabolism of arginine regulates the NO/polyamines balance [27]. The role of polyamines in the physiopathology of PH is unclear. Indeed, while some studies linked spermidine and putrescine with an increase in RVS.P in CH-induced pulmonary vascular (PV) remodeling [31], others demonstrated their anti-inflammatory effects [32] and, therefore, their putative benefit against PH [33]. On the opposite, the critical role of NO for vasodilatation is well-referenced [34]. Numerous studies linked the progression of PH with NO alteration [35,36,37] and demonstrated that the urea cycle played a major role in PH severity [14,38]. Alteration of arginine metabolism leads to regulation of tryptophan metabolism as already described in the literature about PH [11,15]: Indeed, Lewis et al. reported that indoleamine 2,3-dioxygenase (IDO), the heme-containing enzyme metabolizing tryptophan into kynurenine, was over-activated by a decrease in NO as a compensatory vasodilator pathway [12,39] (Figure 4A3,B3). IDO-deficient mice developed exaggerated hypoxic pulmonary vasoconstriction [40]. Moreover, lung mRNA expression of *IDO* was increased in PH models, and IDO-tryptophan metabolites were correlated with RV dysfunction [12]. However, alteration of tryptophan metabolism has specifically been found altered in pulmonary circulation [12], explaining why kynurenate and xanthurenate were not altered in heart tissues in our study. Tryptophan can also be metabolized by tryptophan hydroxylase (TPH), which yields serotonin. Our results demonstrated alterations of serotonin in the plasma of CH-PH rats. Serotonin has been involved in the pathophysiology of PH, contributing to vasoconstriction and vascular remodeling in PH lungs [41,42]. In our study, a decrease in serotonin was observed in the plasma of the carotid artery, suggesting that tryptophan is mainly metabolized by IDO in our model. 

### 4.2. A Balance between Vasoconstriction and Vasodilatation in the Heart

For the first time, we showed stimulation of arginase activity in heart biopsies from PH models, which suggests that vasoconstriction induced by NO depletion could also affect cardiac arterial tone. Moreover, an alteration of purine metabolism was mainly specific to heart tissues (Figure 4(A4,B4)). Previous studies analyzing the role of purine metabolic pathways in PH mostly focused on serum urate, and cardiac alteration of purines has been weakly investigated in this disease [43,44]. Apart from the PH context, cardiac efflux of purine metabolites triggered by ischemia or CH are well referenced [45]. Several studies correlated alteration of purine metabolism with a decrease in functional capacities in patients with cardiac failure [46,47] and deficient oxidative metabolism during hypoxic states [12,13,46]. As an end-product of purine metabolism, allantoin was the most impacted metabolite and the most correlated with RV-PV function parameters, as previously reported [12]. Allantoin has been associated with central antihypertensive effects in rats [48]. Our results suggest that allantoin would be a putative biomarker of cardiac hypoxia during PH. In addition, kynurenine, from the tryptophan pathway, was increased in the heart of CH-PH rats and correlated with PVR, RVS.P, RV weight, and Fulton’s index (Figure 4(A3,B3)). This metabolite has been described as a putative endothelium-derived vasodilator in several models [49]. Thus, our results might suggest a protective role of purine and tryptophan metabolic pathways in response to vasoconstriction in heart of PH rats, suggesting that these pathways could be important for RV remodeling during PH due to hypoxemia.

### 4.3. Polyamine Mediated Cardiac Hypertrophy

We demonstrated that putrescine and acetyl-spermidine were increased in both functional and dysfunctional hypertrophied RV in PH. Indeed, super activated arginase led to a strong increased in polyamines (putrescine, spermidine, and acetyl-spermidine), specifically in the heart of CH and MCT-PH rats and particularly in RV (Figure 4(A1,B1)). This alteration was highly correlated to RVS.P, RV weight, Fulton’s index, and PVR. Yet, polyamines have been known to contribute to pathological processes in the heart, including cardiac hypertrophy [50,51,52,53]. Particularly, the increase in polyamines is mediated by pulmonary artery constriction, enhanced cardiac work consecutive to hypoxic PH, and reduced availability of oxygen after cardiac overload [53]. Moreover, several studies reported that polyamines, and particularly spermidine, are thought to be responsible for the induction of autophagy [54], a critical process involved in cardiac hypertrophy [55]. Furthermore, polyamines can affect the function of many ion channels, including the inward rectifying K^+^ current, NMDA receptor, cyclic nucleotide-gated channels, voltage-gated sodium channel in different cell types [56,57,58]. By interaction with 14-3-3 proteins, increased polyamines may also downregulate TASK-1 channels [59,60,61], which contributes to the reduction in RV cardiomyocytes’ excitability [30]. Moreover, we previously demonstrated that loss of TASK-1 function was a hallmark of RV hypertrophy/dysfunction associated with PH, including in RV from MCT-PH and CH-PH models [62]. Thus, inhibition of these ion channel families by increased polyamines could have profound consequences on RV function, including resting membrane potential, action potential duration, and excitation, which are observed in isolated cardiomyocytes from different PH animal models [30]. Therefore, significant evidences emerge for the role of polyamines in cardiac pathophysiology and point out these metabolites as putative targets for the treatment of cardiovascular diseases [52]. 

### 4.4. Metabolic Alteration Related to Cardioprotection

Pyrimidine metabolism was the second most impaired pathway in our study (Figure 4(A2,B2)), with a major increase in uridine and orotate, as already reported in PH and heart ischemia [12,63]. Due to the high myocardial turnover of pyrimidine metabolites, any alteration in their utilization or supply could have severe metabolic consequences [64]. In case of alteration, cardiac de novo synthesis of pyrimidines is inefficient, and precursors are mainly released by the liver and transferred through the bloodstream [65]. This fact might explain why we detected almost all pyrimidine alterations in both plasma and heart of CH-PH rats. Cardioprotection of pyrimidine metabolites, such as uridine and orotate, has been demonstrated for more than twenty years [63]. An international symposium about the cardioprotective effect of orotate highlighted that this metabolite (i) improves the tolerance of infarcted heart to ischemia, (ii) reduces the severity of chronic myocardial dysfunction, and (iii) improves contractile function in rats with hypertrophying hearts [64]. These effects could be explained by the fact that orotate increases pyrimidines’ base de novo-production in the liver, which consequently activates a metabolic cascade preventing ATP depletion in the myocardium [64,66,67]. We suggest an orotate increase in MCT- and CH-PH rats as a cardioprotective response to the cardiac vasoconstriction and hypertrophy.

**Figure 5:**
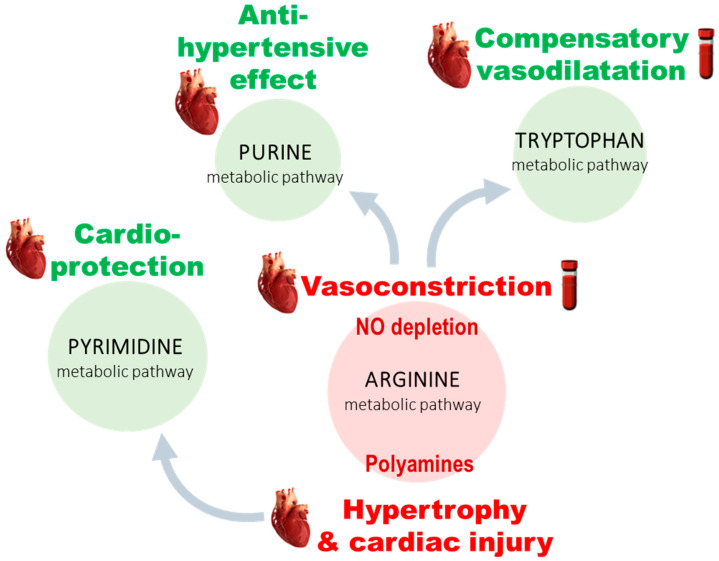
Interpretation of metabolic alterations detected in plasma and heart samples of rat models of PH. (green = increased; red = decreased).

Our results contribute to the understanding of PH pathogenesis as well as RV remodeling. Thanks to metabolomics, some hypotheses were formulated about the communication between four main metabolic pathways (Figure 5). Arginine metabolism is supposed to be central in the disease outbreak through the depletion in NO and, therefore, the induction of vasoconstriction at both peripheral and cardiac levels. Some compensatory reactions are probably promoted to slow down the rise in arterial tone thanks to the stimulation of IDO activity (in plasma and heart) and synthesis of allantoin (specifically in heart). We assume that increased polyamines are involved in the development of cardiac hypertrophy and that cardiac injury is partly offset by several cardioprotective pyrimidines. These different factors stand as putative new therapeutic targets or candidate biomarkers for the early diagnostic of cardiac impairment secondary to PH. Such biomarkers could be detected in blood samples or used as PET tracers when limited to the heart compartment.

### 4.5. Limitations

Using experimental PH animal models allowed myocardium metabolic disturbances to be studied in comparison to plasmatic alterations, but there is no animal model fully mimicking pathological features of humans, and clinical trials are necessary to confirm our observations. Moreover, the current study did not allow gender influence in RV dysfunction to be considered since only male rats were analyzed for their higher sensitivity to experimental PH. In our study, MCT rats gave less homogenous and interpretable results than CH rats. Moreover, the CH model is considered as inducing a weaker intensity of obstructive remodeling and modest hemodynamic abnormalities, which can make the alterations less representative of severe human cardiac remodeling. As we know that a greater proportion of female PAH patients have been consistently observed, the metabolic signature of RV failure could be investigated in females in future studies. As RV and LV tissues are composed of several cell types, including endothelial cells, fibroblasts, cardiomyocytes, inflammatory cells, we plan to analyze the metabolic signature of adult rat ventricular myocytes in different experimental conditions in future studies. At least, the study explored the surface of metabolite change in RV; neither a theory nor validation of these findings was conducted. One should emphasize that metabolomic differences between groups may be due to rat model differences rather than RV phenotype.

In conclusion, this study investigated the metabolomic signature of cardiac remodeling on two PH rat models using a robust untargeted metabolomic platform coupled to an in-house database for the identification of hundreds of metabolites. Predominant alterations of arginine, pyrimidine, purine, and tryptophan metabolic pathways were detected on both plasma and heart tissue samples. These pathways are known to be involved in vasodilatation, cell proliferation, hypertrophy, and cardioprotection in either PH or cardiac ischemia. Here, for the first time, we gave new insights into the organ origin of their alteration and brought new insights about their implication, specifically in cardiac failure during PH. Metabolites from arginine, pyrimidine, tryptophan, and purine pathways that are highly correlated with cardiac clinical parameters were highlighted as candidate biomarkers of RV remodeling or as putative therapeutic targets. These finding open novel research perspectives to find biomarkers for early detection of RV failure in PH, and further clinical trials are needed to confirm our hypotheses in patients.

## Figures and Tables

**Table 1:** Echocardiography and catheterization measures on CH-PH and MCT-PH rats and their respective controls.

		Normoxia (Nx)	CH-PH	Nx vs. CH-PH	Vehicle	MCT-PH	Vhcl vs. MCT-PH	CH-PH vs. MCT-PH
					(Vhcl)			
Morphometric	Body weight (g)	259.8 ± 19.14	228 ± 19.22	**	277.64 ± 22.82	249.27 ± 56.16	ns	*
Parameters	RV weight (mg)	173.6 ± 13.43	381.2 ± 81.79	***	154.33 ± 17.4	268.27 ± 70.6	***	**
	LV + Septum weight (mg)	603.3 ± 56.57	606.6 ± 64.14	ns	595.2 ± 37.4	554.73 ± 111.69	ns	ns
	Fulton’s index	0.29 ± 0.02	0.63 ± 0.12	***	0.26 ± 0.03	0.49 ± 0.11	***	*
	TAPSE (mm)	0.24 ± 0.05	0.26 ± 0.06	ns	0.3 ± 0.01	0.23 ± 0.04	**	ns
RV Parameters	VTI-PA	6.38 ± 0.62	5.65 ± 0.56	*	6.63 ± 0.76	4.29 ± 1.21	***	*
	PA diam (mm)	2.44 ± 0.15	2.26 ± 0.24	ns	2.4 ± 0.2	2.29 ± 0.28	ns	ns
	PAAT (ms)	34.3 ± 3.56	27 ± 3.6	**	32.43 ± 1.71	23.44 ± 4.05	***	ns
	RVET (ms)	78.6 ± 5.88	80.7 ± 5.98	ns	80.71 ± 5.63	76.22 ± 7.04	ns	ns
	Cycle length (ms)	159.1 ± 10.32	160.2 ± 8.6	ns	154.5 ± 21.21	170.44 ± 16.05	ns	ns
	RV free wall thickness (mm)	1.12 ± 0.16	1.36 ± 0.23	*	0.99 ± 0.18	1.33 ± 0.23	**	ns
	RV_FS (%)	47.94 ± 8.16	39.02 ± 11.18	ns	44.13 ± 6.66	32.65 ± 14.76	ns	ns
	VTI-Ao	5.9 ± 0.92	5.57 ± 0.72	ns	8.16 ± 0.91	5.88 ± 1.29	***	ns
LV Parameters	LV free wall thickness (mm)	1.53 ± 0.24	1.85 ± 0.24	*	1.64 ± 0.17	1.48 ± 0.17	ns	**
	LV_FS (%)	49.16 ± 8.79	48.11 ± 8.21	ns	41.47 ± 6.8	48.89 ± 8.13	ns	ns
Closed Chest Heart	RVS.P (mmHg)	34.1 ± 6.7	68.26 ± 10.83	***	32.83 ± 4.01	89.46 ± 26.02	***	*
Catheterization	HR (beat/min)	346.6 ± 48.09	376.4 ± 43.29	ns	346.62 ± 43.26	333.38 ± 39.75	ns	ns
	MaxdPdt (mmHg/s)	2629.61 ± 1444.89	4448.63 ± 1990.74	*	3422.57 ± 627.95	6913.86 ± 1317.87	***	*
	MindPdt (mmHg/s)	−1095.54 ± 428.09	−2265.96 ± 921.23	**	−1385.43 ± 306.83	−2194.57 ± 568.54	***	ns
	CO (ml/min)	81.55 ± 3.82	72.56 ± 16.57	ns	85.35 ± 14.13	53 ± 18.84	***	*
	PVR	0.44 ± 0.06	0.98 ± 0.19	***	0.4 ± 0.09	1.97 ± 1.14	***	*
	EDP (mmHg)	4.92 ± 3.02	12.79 ± 11.53	*	4.68 ± 1.96	17.34 ± 7.72	***	ns
	RV Contractility index (s^−1^)	123.9 ± 26.46	121.7 ± 13.63	ns	122.08 ± 28.22	75.25 ± 14.7	***	***

Data are presented as mean ± standard deviation. *t*-tests were used after verification of normal distribution of values (Shapiro–Wilk normality test). Significance: * *p ≤* 0.05; ** *p ≤* 0.01; *** *p ≤* 0.001. ns: Non-significant. pulmonary artery acceleration time (PAAT, ms), pulmonary artery and aorta velocity time integral (VTI-PA and VTI-Ao), Tricuspid annular plane systolic excursion (TAPSE; mm), RV ejection time (RVET; ms), Cycle length (ms), RV and LV fractional shortening (RV FS and LV FS; %), right ventricle systolic pressure (RVS.P, mmHg), heart rate (HR, bpm), cardiac output (CO, mL/min), pulmonary vascular resistance (PVR, WU), end diastolic pressure (EDP, mmHg).

**Table 2:** Discriminant metabolites from arginine, pyrimidine, purine, and tryptophan pathways. ns: Non-significant. nr: not detected or not robust feature. ^a^ Identification validated by MS/MS experiments. Significance: * *p ≤* 0.05 ** *p ≤* 0.01 *** *p ≤* 0.001. ns: Not significant. RV: Right ventricle. LV: Left ventricle. FC: Fold Change (in green increased metabolites, in red decreased metabolites).

		Fold Change (PH/CTRL) and Significance					
		CH vs. Normoxia			MCT vs. Vehicle		
		Plasma	RV	LV	Plasma	RV	LV
Arginine metabolic pathway	Spermidine ^a^	ns	1.50 *	1.27 ***	ns	1.33 **	ns
	Acetyl-spermidine ^a^	ns	2.08 ***	1.80 ***	ns	2.97 ***	2.02 **
	Putrescine ^a^	ns	1.87 ***	1.67 ***	ns	1.75 ***	ns
	Guanidinoacetate ^a^	1.68 ***	3.01 ***	1.97 ***	0.74 *	1.54 *	ns
	Arginine ^a^	0.75 *	0.85 *	0.81 ***	0.76 **	0.85 **	ns
	Creatine ^a^	0.72 **	0.79 ***	0.77 ***	ns	0.88 **	0.91 ***
	Fumarate ^a^	1.55 *	ns	1.23 ***	ns	1.43 *	ns
	Ornithine ^a^	1.34 ***	ns	ns	ns	1.57 **	ns
	Creatinine ^a^	1.15 ***	ns	ns	1.17 **	ns	ns
	Aspartate ^a^	1.40 **	1.26 *	ns	ns	ns	ns
	Oxoglutarate ^a^	1.56 ***	0.75 *	ns	ns	ns	ns
	Guanidinobutanoate ^a^	1.94 ***	ns	1.15 *	ns	ns	ns
	N-acetyl-glutamate ^a^	1.58 **	ns	ns	ns	ns	1.5 *
	Glutamine ^a^	1.36 ***	ns	ns	0.89 *	0.86 **	ns
	Citrulline ^a^	ns	ns	0.88 *	0.87 *	0.85 *	0.80 **
	Aminobutanoate ^a^	ns	ns	0.89 *	ns	ns	ns
	Glutamate ^a^	ns	ns	0.90 *	ns	ns	ns
	Trans-4-L-hydroxy-proline ^a^	1.1 *	ns	0.85 *	ns	ns	ns
Pyrimidine metabolic pathway	Orotate ^a^	1.87 **	2.13 ***	2.05 ***	ns	2.18 **	ns
	Pseudouridine ^a^	1.20 ***	1.27 **	1.19 *	1.09 *	1.28 **	1.29 *
	Uridine ^a^	1.50 *	1.25 *	ns	ns	1.26 *	ns
	Uracil ^a^	1.31 *	ns	1.25 **	1.76 *	1.28 **	ns
	Thymidine ^a^	1.60 ***	ns	ns	1.45 **	1.25 *	ns
	Thymine ^a^	1.30 **	ns	ns	1.51 *	1.47 **	ns
	Deoxyuridine ^a^	1.39 ***	ns	1.24 ***	ns	1.41 *	ns
	Dihydrouracil ^a^	1.28 **	ns	ns	1.66 **	ns	ns
	Cytosine ^a^	1.27 **	1.19 **	1.18 *	ns	1.19 *	ns
	Deoxycytidine ^a^	1.32 ***	1.20 *	1.28 **	ns	ns	ns
	Acetyl-cytidine ^a^	ns	ns	ns	1.37 *	1.64 **	1.49 *
	5-Methyldeoxycytidine ^a^	ns	1.45 *	1.55 **	ns	1.59 **	ns
	Dihydroorotate	ns	ns	ns	1.60 *	ns	ns
Purine metabolism	Allantoin ^a^	1.21 **	1.24 *	1.39 ***	1.21 *	1.26 **	1.34 *
	7-Methyl-guanine ^a^	1.18 *	1.23 *	1.24 **	ns	1.46 **	1.51 *
	Methyl-inosine ^a^	1.23 *	ns	ns	ns	1.46 *	ns
	Xanthosine ^a^	nr	1.38 *	ns	nr	1.90 **	ns
	Xanthine ^a^	nr	ns	1.12 *	nr	1.18 *	ns
	Inosine ^a^	nr	ns	0.92 ***	nr	0.90 **	0.94 *
	Guanosine ^a^	nr	ns	0.85 *	nr	0.71 ***	0.82 *
	Adenine ^a^	nr	0.67 *	ns	nr	ns	ns
	5’AMP ^a^	nr	1.18 *	ns	nr	ns	ns
	Urate ^a^	ns	ns	1.28 *	2.50 *	1.8 *	ns
Tryptophan metabolic pathway	Indole-propionate ^a^	1.60 *	1.81 *	2.11 **	ns	ns	ns
	Indolelactate ^a^	0.55 ***	0.65 *	ns	ns	ns	ns
	Kynurenine ^a^	1.28 *	ns	1.34 *	ns	ns	ns
	Tryptophan ^a^	ns	ns	1.15 *	ns	1.32 **	ns
	Tyrosine ^a^	ns	ns	ns	1.33 *	1.34 **	1.21 *
	Phenylalanine ^a^	0.82 ***	ns	ns	ns	1.28 *	ns
	Acetyl-serotonin	ns	ns	ns	0.57 **	1.54 **	1.88 *
	Serotonin	0.63 *	nr	nr	ns	nr	nr
	Xanthurenate ^a^	ns	nr	nr	1.39 *	nr	nr
	Aminophenol	ns	ns	ns	0.59 *	ns	ns
	Kynurenate ^a^	ns	ns	ns	1.58 *	ns	ns
	N-acetyl-tryptophan ^a^	ns	ns	ns	1.51 *	ns	2.47 *

**Table 3:** Spearman correlations between metabolites from arginine, pyrimidine, purine, and tryptophan and five catheterization and echocardiography parameters. RT: Retention time in chromatographic separation. PL: Plasma. RV: Right ventricle. LV: LEFT ventricle. Significance: * *p ≤* 0.05 ** *p ≤* 0.01 *** *p ≤* 0.001. ns: non-significant. nr: Not detected or not robust feature.

				SPEARMAN CH-PH vs. Normoxia															SPEARMAN MCT-PH vs. Vehicle														
				RVS.P			RV Weight			Fulton’s Index			PVR			PAAT			RVS.P			RV weight			Fulton’s Index			PVR			PAAT		
Name	*m/z*	RT (min)	Ion	PL	RV	LV	PL	RV	LV	PL	RV	LV	PL	RV	LV	PL	RV	LV	PL	RV	LV	PL	RV	LV	PL	RV	LV	PL	RV	LV	PL	RV	LV
Arginine	175.1187	0.7	[M+H]^+^	−0.51 *	−0.53 *	−0.74 ***	−0.48 *	ns	−0.72 ***	ns	ns	−0.63 **	ns	ns	−0.81 ***	ns	ns	0.51 *	−0.54 *	−0.54 **	ns	ns	−0.42 *	ns	−0.53 **	−0.58 **	ns	−0.46 *	−0.51 *	ns	0.66 ***	0.43 *	ns
Creatine	130.0623	7.6	[M−H]^−^	−0.66 **	−0.76 ***	−0.81 ***	−0.7 ***	−0.71 ***	−0.85 ***	−0.67 **	−0.64 **	−0.79 ***	−0.51 *	−0.76 ***	−0.86 ***	0.52 *	0.69 ***	0.75 ***	ns	−0.44 *	−0.66 **	ns	−0.58 **	−0.7 ***	ns	−0.61 **	−0.73 ***	ns	−0.47 *	−0.75 ***	ns	ns	0.7 ***
Guanidinoacetate	116.0466	8.3	[M−H]^−^	0.69 ***	0.79 ***	0.77 ***	0.71 ***	0.87 ***	0.78 ***	0.7 ***	0.85 ***	0.73 ***	0.65 **	0.83 ***	0.82 ***	ns	−0.76 ***	−0.75 ***	ns	0.47 *	ns	ns	0.63 ***	0.4 *	ns	0.47 *	ns	ns	0.57 **	ns	0.44 *	ns	ns
Aspartate	132.0302	8.20	[M−H]^−^	0.54 *	0.55 *	0.54 *	0.69 ***	0.56 *	0.45 *	0.59 **	0.54 *	0.47 *	0.73 ***	0.51 *	ns	−0.5 *	ns	ns	ns	0.56 **	ns	ns	0.4 *	ns	ns	ns	ns	ns	0.46 *	ns	ns	ns	0.48 *
Fumarate	115.0035	8.8	[M−H]^−^	0.51 *	ns	0.56 **	0.47 *	0.45 *	0.61 **	ns	ns	0.55 *	0.6 **	0.55 *	0.75 ***	ns	ns	−0.57 **	ns	0.64 **	ns	ns	0.53 **	ns	ns	0.53 **	ns	ns	0.66 **	ns	ns	ns	ns
Oxoglutarate	145.0140	8.6	[M−H]^−^	0.66 **	ns	ns	0.69 **	−0.46 *	ns	0.64 **	−0.5 *	ns	0.76 ***	−0.59 **	ns	−0.52 *	0.44 *	ns	ns	ns	ns	ns	ns	ns	ns	ns	ns	ns	ns	ns	ns	ns	ns
N-acetyl-glutamate	188.0563	8	[M−H]^−^	0.53 *	ns	ns	0.5 *	ns	ns	ns	−0.52 *	ns	0.48 *	−0.48 *	ns	ns	ns	ns	ns	ns	ns	ns	ns	ns	ns	ns	ns	ns	ns	ns	ns	ns	ns
Spermidine	146.1651	0.6	[M+H]^+^	ns	0.52 *	0.79 ***	0.5 *	0.6 **	0.75 ***	ns	0.57 **	0.82 ***	0.6 **	0.57 *	0.83 ***	ns	ns	−0.59 **	ns	0.6 **	ns	0.44 *	0.5 *	ns	0.43 *	0.64 ***	ns	ns	0.66 **	ns	ns	−0.64 ***	−0.42 *
Acetyl-spermidine	188.1755	0.7	[M+H]^+^	ns	0.74 ***	0.73 ***	ns	0.82 ***	0.74 ***	ns	0.79 ***	0.78 ***	ns	0.8 ***	0.84 ***	ns	−0.66 **	−0.68 **	ns	0.63 **	0.61 **	ns	0.69 ***	0.54 **	ns	0.83 ***	0.77 ***	ns	0.69 ***	0.69 ***	ns	−0.78 ***	−0.68 ***
Glutamine	145.0616	7.7	[M−H]^−^	0.70 ***	ns	ns	0.6 **	ns	ns	0.74 ***	ns	ns	0.6 **	ns	ns	−0.69 ***	ns	ns	ns	ns	ns	ns	−0.44 *	ns	ns	−0.58 **	ns	ns	−0.48 *	ns	ns	0.48 *	ns
Citrulline	174.088	8.1	[M−H]^−^	ns	ns	−0.52 *	ns	ns	ns	ns	ns	ns	ns	ns	ns	ns	ns	0.46 *	ns	ns	ns	ns	ns	ns	ns	ns	−0.51 **	ns	ns	ns	ns	ns	0.46 *
Guanidinobutanoate	146.0922	0.9	[M+H]^+^	0.82 ***	ns	ns	0.85 ***	ns	ns	0.78 ***	0.46 *	0.57 *	0.85 ***	ns	ns	−0.55 *	ns	ns	ns	ns	ns	ns	ns	ns	ns	ns	ns	ns	ns	ns	ns	ns	ns
Putrescine	89.1072	0.7	[M+H]^+^	ns	0.80 ***	0.78 ***	ns	0.76 ***	0.69 ***	ns	0.76 ***	0.79 ***	ns	0.82 ***	0.79 ***	ns	−0.7 ***	−0.72 ***	ns	0.56 **	ns	ns	0.56 **	ns	ns	0.72 ***	ns	ns	0.58 **	ns	ns	−0.47 *	ns
Creatinine	112.0516	3.5	[M−H]^−^	0.62 **	ns	ns	0.6 **	ns	ns	0.7 ***	ns	ns	0.73 ***	ns	ns	−0.53 *	ns	ns	ns	ns	ns	0.46 *	ns	ns	0.55 **	ns	ns	ns	ns	ns	−0.54 **	ns	ns
Ornithine	131.083	9.6	[M−H]^−^	0.56 *	ns	ns	0.5 *	ns	ns	0.69 ***	ns	ns	0.65 **	ns	ns	−0.49 *	ns	ns	ns	0.77 ***	ns	ns	0.75 ***	ns	ns	0.64 ***	ns	ns	0.81 ***	ns	ns	−0.46 *	ns
Aminobutanoate	102.0561	8.4	[M−H]^−^	ns	−0.48 *	ns	ns	ns	−0.54 *	ns	ns	−0.56 *	ns	−0.49 *	ns	ns	0.45 *	0.59 **	ns	ns	ns	ns	ns	ns	ns	ns	ns	ns	ns	ns	ns	ns	ns
Glutamate	146.0455	8.3	[M−H]^−^	ns	ns	ns	ns	ns	−0.55 *	ns	ns	−0.57 **	ns	ns	ns	ns	ns	0.59 **	ns	ns	ns	ns	ns	ns	ns	ns	ns	ns	ns	ns	ns	ns	ns
Trans-4-L-hydroxy-proline	130.0509	7.8	[M−H]^−^	ns	ns	ns	ns	ns	ns	ns	ns	ns	ns	ns	ns	ns	ns	0.68 **	ns	ns	ns	ns	ns	ns	−0.45 *	ns	ns	ns	ns	ns	ns	ns	0.43 *
Orotate	155.0097	4.8	[M−H]^−^	0.69 ***	0.81 ***	0.75 ***	0.7 ***	0.9 ***	0.76 ***	0.57 *	0.82 ***	0.73 ***	0.8 ***	0.9 ***	0.83 ***	−0.65 **	−0.72 ***	−0.63 **	ns	0.58 **	ns	ns	ns	ns	ns	0.54 **	ns	ns	0.64 **	ns	ns	−0.46 *	ns
Cytosine	112.0503	0.9	[M+H]^+^	0.78 ***	0.53 *	0.55 *	0.83 ***	0.67 **	0.51 *	0.56 *	0.55 *	ns	0.85 ***	0.62 **	0.5 *	−0.52 *	−0.68 **	ns	ns	ns	ns	ns	ns	ns	ns	ns	ns	ns	0.46 *	ns	ns	ns	ns
Deoxycytidine	228.0975	0.9	[M+H]^+^	0.80 ***	0.5 *	0.71 ***	0.87 ***	0.61 **	0.71 ***	0.63 **	0.46 *	0.52 *	0.89 ***	0.66 **	0.73 ***	−0.59 **	ns	ns	ns	ns	−0.52 *	ns	ns	ns	ns	ns	ns	ns	ns	ns	ns	ns	ns
Deoxyuridine	227.0672	2.8	[M−H]^−^	0.74 ***	ns	0.47 *	0.69 ***	ns	0.48 *	0.68 **	ns	ns	0.8 ***	ns	0.47 *	ns	ns	ns	ns	0.6 **	ns	ns	0.69 ***	0.54 **	ns	0.62 ***	0.5 *	ns	0.59 **	ns	ns	−0.49 *	ns
Pseudouridine	243.0619	5.8	[M−H]^−^	0.56 *	0.51 *	0.51 *	0.59 **	0.58 **	0.49 *	0.65 **	0.5 *	ns	0.68 **	0.61 **	0.52 *	−0.49 *	−0.46 *	ns	ns	0.7 ***	ns	0.41 *	0.48 *	ns	0.45 *	0.62 ***	0.49*	ns	0.67 ***	ns	ns	−0.67 ***	ns
Uridine	243.0618	3.8	[M−H]^−^	0.57 **	ns	ns	0.48 *	ns	ns	ns	ns	ns	0.59 **	ns	ns	ns	ns	ns	ns	0.5 *	ns	ns	0.66 ***	ns	ns	0.62 ***	ns	ns	0.46 *	ns	ns	ns	ns
Thymidine	243.0971	1.9	[M+H]^+^	0.74 ***	ns	ns	0.78 ***	ns	ns	0.63 **	ns	ns	0.81 ***	ns	ns	−0.57 **	ns	ns	ns	0.6 **	ns	0.45 *	ns	ns	0.61 **	0.47 *	ns	ns	0.57 **	ns	−0.46 *	−0.63 ***	ns
Thymine	125.036	2.3	[M−H]^−^	0.65 **	ns	ns	0.66 **	ns	ns	0.64 **	ns	ns	0.58 *	ns	ns	ns	ns	ns	0.46 *	0.66 ***	ns	0.47 *	0.4 *	ns	0.62 ***	0.58 **	ns	ns	0.62 **	ns	−0.58 **	−0.64 ***	ns
Uracil	111.0198	2.9	[M−H]^−^	0.48 *	ns	0.61 **	0.48 *	ns	0.54 *	ns	ns	0.45 *	0.51 *	ns	0.5 *	ns	ns	ns	ns	0.67 ***	ns	ns	0.56 **	ns	0.45 *	0.53 **	ns	ns	0.71 ***	ns	−0.62 **	−0.58 **	ns
5-methyldeoxycytidine	242.1132	1	[M+H]^+^	ns	0.45 *	0.6 **	ns	0.46 *	0.52 *	ns	0.55 *	0.52 *	ns	0.46 *	0.6 **	ns	ns	−0.49 *	0.55 **	0.63 **	ns	ns	0.42 *	ns	ns	0.42 *	ns	ns	0.73 ***	ns	ns	−0.59 **	ns
Acetyl-cytidine	286.103	2	[M+H]^+^	ns	ns	ns	ns	ns	ns	ns	ns	ns	ns	ns	ns	ns	ns	ns	0.51 *	0.6 **	ns	ns	ns	ns	ns	0.59 **	0.46 *	0.53 *	0.66 **	ns	−0.49 *	−0.72 ***	−0.46 *
Dihydrouracil	115.0502	0.8	[M+H]^+^	0.62 **	−0.52 *	−0.46 *	0.69 **	−0.56 *	−0.61 **	0.47 *	−0.49 *	−0.59 **	0.65 **	−0.57 *	−0.56 *	ns	0.79 ***	ns	0.6 **	−0.45 *	ns	0.47 *	ns	ns	0.65 ***	ns	ns	0.53 *	−0.51 *	ns	−0.55 **	ns	ns
Dihydroorotate	157.0253	5.5	[M−H]^−^	ns	−0.52 *	ns	ns	−0.56 *	ns	ns	−0.49 *	ns	ns	−0.57 *	ns	ns	0.79 ***	ns	0.53 *	−0.45 *	ns	0.42 *	ns	ns	0.51 **	ns	ns	0.56 **	−0.51 *	ns	−0.75 ***	ns	ns
Allantoin	157.0364	7.2	[M−H]^−^	0.69 ***	0.53 *	0.80 ***	0.68 **	0.58 **	0.81 ***	0.5 *	0.5 *	0.74 ***	0.72 ***	0.55 *	0.85 ***	ns	ns	−0.61 **	ns	0.64 **	0.53 *	ns	ns	ns	ns	0.49 *	0.57 **	ns	0.71 ***	0.61 **	−0.55 **	−0.66 ***	−0.46 *
7-methyl-guanine	164.0579	3.5	[M−H]^−^	ns	ns	0.68 **	0.45 *	0.51 *	0.67 **	0.57 *	0.48 *	0.52 *	0.49 *	ns	0.71 ***	ns	ns	−0.53 *	ns	0.65 ***	0.43 *	ns	0.44 *	ns	ns	0.58 **	0.53 **	ns	0.63 **	ns	ns	−0.57 **	ns
Methyl-inosine	283.1033	1.7	[M+H]^+^	0.65 **	ns	ns	0.52 *	ns	ns	ns	ns	ns	0.56 *	ns	ns	ns	ns	ns	ns	0.68 ***	ns	ns	0.41 *	ns	ns	0.49 *	ns	ns	0.66 **	ns	ns	−0.47 *	ns
Inosine	269.0876	1.2	[M+H]^+^	nr	ns	−0.63 **	nr	ns	−0.7 ***	nr	ns	−0.83 ***	nr	ns	−0.65 **	nr	ns	0.65 **	nr	−0.58 **	ns	nr	−0.57 **	ns	nr	−0.65 ***	ns	nr	−0.59 **	ns	nr	ns	0.46 *
Guanosine	282.0841	6	[M−H]^−^	nr	ns	ns	nr	ns	ns	nr	ns	ns	nr	ns	ns	nr	0.57 **	ns	nr	ns	ns	nr	−0.51 **	ns	nr	−0.57 **	ns	nr	ns	−0.46 *	nr	0.49 *	ns
Xanthosine	283.0679	6.4	[M−H]^−^	nr	ns	ns	nr	ns	ns	nr	0.46 *	ns	nr	0.51 *	ns	nr	−0.46 *	ns	nr	0.51 *	ns	nr	0.52 **	ns	nr	0.61 **	ns	nr	0.52 *	ns	nr	−0.65 ***	ns
Xanthine	153.0405	1	[M+H]^+^	nr	ns	ns	nr	ns	ns	nr	ns	ns	nr	0.49 *	ns	nr	−0.51 *	−0.46 *	nr	ns	ns	nr	ns	ns	nr	0.39 *	ns	nr	ns	ns	nr	−0.51 *	ns
Urate	169.0352	1	[M+H]^+^	ns	ns	ns	ns	ns	0.49 *	ns	ns	0.53 *	ns	ns	0.65 **	ns	ns	ns	ns	ns	ns	ns	0.46 *	ns	ns	0.51 **	ns	ns	ns	ns	ns	−0.42 *	ns
Adenine	136.0616	0.9	[M+H]^+^	nr	−0.62 **	ns	nr	−0.54 *	ns	nr	−0.59 **	ns	nr	ns	ns	nr	0.51 *	ns	nr	ns	ns	nr	ns	ns	nr	ns	ns	nr	ns	ns	nr	ns	ns
5’-AMP	348.0697	0.9	[M+H]^+^	nr	−0.51 *	ns	nr	−0.49 *	ns	nr	ns	ns	nr	−0.49 *	ns	nr	0.71 ***	ns	nr	ns	ns	nr	ns	ns	nr	ns	ns	nr	ns	ns	nr	ns	ns
Indole-propionate	188.0714	2.3	[M−H]^−^	ns	ns	0.5 *	ns	0.49 *	0.58 **	ns	ns	0.6 **	ns	0.47 *	0.67 **	ns	ns	−0.57 **	ns	0.49 *	ns	ns	ns	ns	ns	ns	ns	ns	0.49 *	0.46 *	ns	ns	ns
Indolelactate	204.0664	2.8	[M−H]^−^	−0.68 ***	−0.49 *	ns	−0.64 **	ns	ns	−0.57 *	ns	ns	−0.52 *	ns	ns	0.66 **	0.48 *	ns	−0.43 *	ns	ns	ns	ns	ns	ns	ns	ns	ns	ns	ns	ns	ns	ns
Kynurenine	209.0918	1.9	[M+H]^+^	0.63 **	ns	0.61 **	0.7 ***	ns	0.63 **	0.64 **	ns	0.54 *	0.74 ***	ns	0.69 **	ns	ns	ns	ns	ns	ns	ns	ns	ns	ns	ns	ns	ns	ns	ns	ns	ns	ns
Tryptophan	203.0822	4.5	[M−H]^−^	ns	ns	0.5 *	ns	ns	0.5 *	ns	ns	0.45 *	ns	ns	0.53 *	ns	ns	−0.54 *	ns	0.5 *	ns	−0.44 *	ns	ns	−0.6 **	ns	ns	ns	0.49 *	ns	0.58 **	ns	ns
Tyrosine	182.0808	1.1	[M+H]^+^	ns	ns	ns	ns	ns	ns	ns	ns	ns	ns	ns	ns	ns	ns	ns	0.64 **	0.5 *	ns	0.56 **	ns	ns	0.48 *	ns	ns	0.68 ***	0.53 *	0.59 **	−0.46 *	−0.56 **	ns
Phenylalanine	164.0714	3.2	[M−H]^−^	−0.81 ***	ns	ns	−0.8 ***	ns	ns	−0.7 ***	ns	ns	−0.76 ***	ns	ns	0.7 ***	ns	ns	ns	0.53 *	ns	ns	ns	ns	ns	0.43 *	ns	ns	0.48 *	ns	ns	−0.58 **	ns
Acetyl-serotonin	219.1127	5.1	[M+H]^+^	ns	ns	ns	ns	ns	ns	ns	ns	ns	ns	ns	ns	ns	ns	ns	ns	0.44*	ns	−0.4 *	0.54 **	ns	−0.50 **	0.61 **	ns	ns	ns	0.45 *	0.65 ***	ns	ns
Serotonin	160.0755	1.5	[M+H−NH3]^+^	−0.58 **	nr	nr	−0.51 *	nr	nr	−0.6 **	nr	nr	ns	nr	nr	0.52 *	nr	nr	−0.44 *	nr	nr	ns	nr	nr	−0.42 *	nr	nr	−0.44 *	nr	nr	0.71 ***	nr	nr
Xanthurenate	206.0447	4.1	[M+H]^+^	ns	nr	nr	ns	nr	nr	ns	nr	nr	ns	nr	nr	ns	nr	nr	ns	nr	nr	ns	nr	nr	ns	nr	nr	ns	nr	nr	−0.48 *	nr	nr
Aminophenol	110.0598	1	[M+H]^+^	ns	ns	ns	ns	ns	ns	ns	ns	ns	ns	ns	ns	ns	0.54 *	ns	ns	ns	ns	ns	ns	ns	−0.49 *	ns	ns	−0.47 *	ns	ns	0.56 **	ns	ns
Kynurenate	190.0496	4.5	[M+H]^+^	ns	ns	ns	ns	ns	ns	ns	ns	ns	ns	ns	ns	ns	ns	ns	ns	ns	ns	ns	ns	ns	ns	ns	ns	ns	ns	ns	ns	ns	ns
N-acetyl-tryptophan	247.1074	6	[M+H]^+^	ns	ns	ns	ns	ns	ns	ns	ns	ns	ns	ns	ns	ns	ns	ns	0.72 ***	ns	ns	0.59 **	ns	ns	0.5 **	ns	ns	0.62 **	ns	ns	ns	ns	ns
	**Caption**			−0.6 < R or R > 0.6				−0.7 < R or R > 0.7				−0.8 < R or R > 0.8																					

## Data Availability

Row data were submitted for paper review process and are available on demand via the corresponding author.

## Supplementary Materials

The following are available online at https://www.mdpi.com/article/10.3390/cells10061559/s1, Figure S1: Representative echocardiographic illustrations allowing us to measure all parameters presented in the study, Figure S2: Multivariate analyses on metabolomic analyses of CH-PH and MCT-PH sample sets, Figure S3: Arginine over ornithine ratios and arginine over guanidinoacetate ratios in CH-PH and MCT-PH rats and their respective controls. Table S1: Summary of metabolomic analyses of plasma and heart of CH-PH and MCT-PH rats.
