# Supplementary material for: Right Ventricle Remodeling Metabolic Signature in Experimental Pulmonary Hypertension Models of Chronic Hypoxia and Monocrotaline Exposure
Source: Cells. 2021 Jun 21;10(6):1559. doi: 10.3390/cells10061559 (PMC8235667; doi:10.3390/cells10061559)
Supplement: Supplementary file 1 [file cells-10-01559-s001.zip › cells-1232914-SI.pdf]

cells MDPI

Detection of metabolomic signature of PH-induced cardiac remodeling.

Metabolomic analyses on plasma, RV and LV of CH-PH and MCT-PH biopsies sets led to the detection of ~5000 analytically relevant metabolite features on average using a ZICpHILIC column with an MS detection in the negative ionization mode (HILIC(-) conditions) and ~8000 using a C18 column in the positive ionization mode (C18(+) conditions) (Supplementary Table 1). After data filtering (see Method), about 500 features from the C18(+) and about 250 from the HILIC(-) analysis matched the accurate mass and retention time of the metabolites included in our chemical database (about 60 of which were in common). Eliminating MS signal redundancy (e.g. adduct, fragment ions and isotopes) and merging C18(+) and HILIC(-) datasets returned 294, 280 and 311 annotated unique metabolites in plasma, RV and LV biopsies, respectively (Supplementary Excel file Table 2).

Statistical analysis

Multivariate statistical analyses showed that metabolomics allowed separation of CH-PH and MCT-PH rats from their respective control groups. CH-PH rats were separated from their controls in PCA analyses and clear separation were get in OPLS-DA with $pQ2<0.01$ and $Q2Y>0.80$ for every organ and analysis (Supplementary Figure 1). Metabolites showing both $VIP>1$ and $p\text{-value}\leq 0.05$ were considered significant. As a result, 85 and 38 annotated metabolites were significant in plasma of CH-PH and MCT-PH rat models, respectively. In RV tissues, 60 and 54 annotated metabolites proved statistically relevant for CH-PH and MCT-PH, respectively. LV tissues analyses showed 71 and 27 significant annotated metabolites for CH-PH and MCT-PH rats respectively (Supplementary Excel file Table 2).

Table S1. Summary of metabolomic analyses of plasma and heart of CH-PH and MCT-PH rats. Number of detected, annotated and significant (Wilcoxon test with $p\leq 0.05$) robust features in Hilic negative and C18 positive analytical modes.

"recovery Hilic/C18" indicates features that were detected and equally annotated in both Hilic Neg and C18 Pos modes. One given metabolite can be represented by several features named "annotated features" such as adducts, fragments and isotopologues ions. "Annotated metabolites" indicates that we considered only on feature per metabolites (monoisotopic ions or most intense adduct ions).

		**CH-PH rats**						**MCT-PH rats**					
		**Plasma**		**Right ventricle**		**Left ventricle**		**Plasma**		**Right ventricle**		**Left ventricle**	
		**Hilic Neg**	**C18 Pos**	**Hilic Neg**	**C18 Pos**	**Hilic Neg**	**C18 Pos**	**Hilic Neg**	**C18 Pos**	**Hilic Neg**	**C18 Pos**	**Hilic Neg**	**C18 Pos**
Detection Filtering	Total detected features	5025	7349	4482	6844	5014	9730	5018	7360	4471	6832	5045	9703
	Robust features	2632	2469	2473	2775	3157	3744	2673	2486	2475	2773	3155	3742
Annotation	Annotated feature	455	252	509	232	606	255	455	252	509	232	606	255
	Annotated feature (considering recovery Hilic/C18)	706		684		796		706		684		796	
	Annotated metabolites (considering recovery Hilic/C18)	294		280		311		294		280		311	
Significanc e	Significant and annotated features	256	110	155	81	217	96	79	53	148	74	65	44
	Significant and annotated metabolites	110	38	61	35	44	41	36	30	60	31	21	19
	**Significant and annotated metabolites** (considering recovery Hilic/C18)	**85**		**60**		**71**		**38**		**54**		**27**	

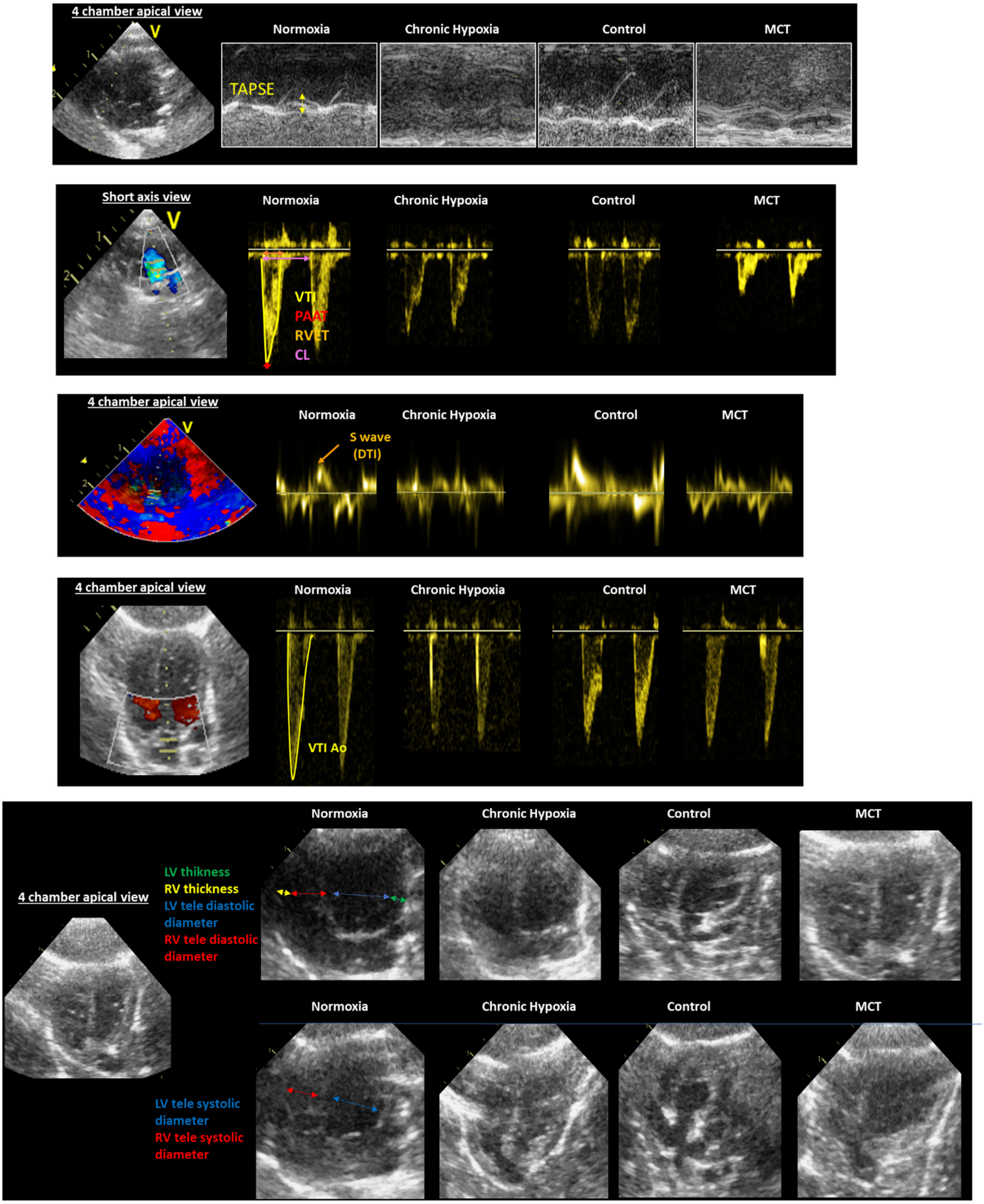

Figure S1. *Representative echocardiographic illustrations allowing us to measure all parameters presented in the study.* All analyses were performed in a blinded manner: rats' experimental conditions were unknown by the operator during TTE examination and data interpretation. In parasternal short axis view we measured pulmonary artery acceleration time (PAAT; ms), pulmonary artery velocity time integral (VTI-PA), RV ejection time (RVET; ms), Cycle length (ms) were performed as previously described (17). In the 4-cavity view performed in the subcostal way, we measured ascending aorta velocity time integral (VTI-Ao) reflecting LV cardiac output, Tricuspid annular plane systolic excursion (TAPSE; mm), RV and LV

thickness (mm). RV and LV fractional shortening (RV FS and LV FS; %) correspond to the percent change in LV and RV cavity diameters respectively. LV FS = ((LV EDd-LV EDs/LV EDd)*100 or RV FS = (RV EDd-RV EDs/RV EDd)*100. .

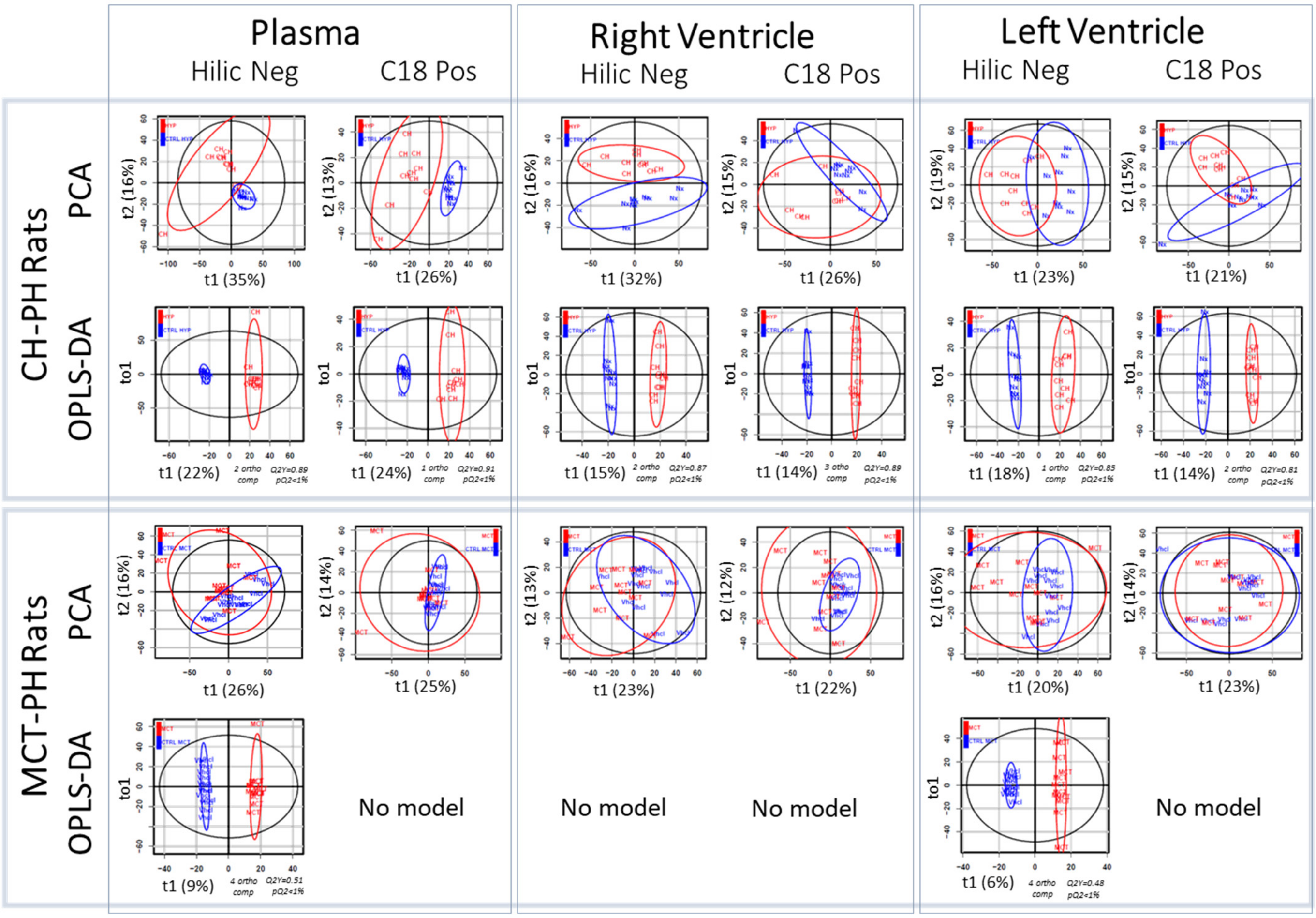

Figure S2. Multivariate analyses on metabolomic analyses of CH-PH and MCT-PH sample sets. PCA and OPLS-DA were performed on all robust features detected in plasma, right ventricle and left ventricle of CH-PH rats "CH" (red), MCT-PH rats "MCT" (red), normoxia rats "Nx" as controls for CH-PH rats (blue) and vehicle rats "Vhcl" as controls for MCT-PH rats (blue). "ortho comp" = orthogonal component. Validating test on 100 permutations. OPLS-Da validated when pQ2≤1% and Q2Y>0.5.

ARG/ORN	**Normoxia (Nx)**	**CH-PH**	**Nx vs CH-PH p-value**	**Vehicle (Vhcl)**	**MCT-PH**	**Vhcl vs MCT-PH p-value**

**Plasma**	1.20 ± 0.13	0.72 ± 0.28	0.0001	1.47 ± 0.63	0.96 ± 0.38	0.02
**RV**	17.73 ± 5.10	13.40 ± 4.15	0.02	21.02 ± 4.06	12.63 ± 4.58	<0.0001
**LV**	13.09 ± 2.48	10.24 ± 1.56	0.006	17.28 ± 4.83	14.46 ± 3.29	0.12

ARG/GUA	**Normoxia (Nx)**	**CH-PH**	**Nx vs CH-PH p-value**	**Vehicle (Vhcl)**	**MCT-PH**	**Vhcl vs MCT-PH p-value**
**Plasma**	16.92 ± 4.77	7.30 ± 1.66	<0.0001	20.12 ± 10.67	16.19 ± 3.58	0.46
**RV**	230.40 ± 66.33	70.18 ± 23.97	<0.0001	157.40 ± 24.03	136.35 ± 159.56	0.0094
**LV**	136.20 ± 36.32	53.96 ± 6.64	<0.0001	125.34 ± 28.14	124.87 ± 61.28	0.43

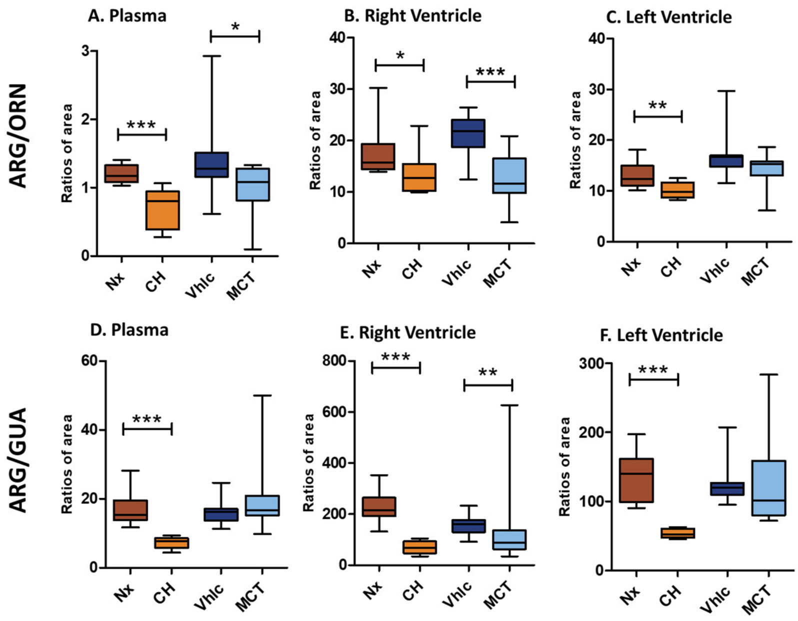

Figure S3. Arginine over ornithine ratios and arginine over guanidinoacetate ratios in CH-PH and MCT-PH rats and their respective controls. Data are presented as mean± standard deviation. t-test were used after verification of normality of distribution of values (Shapiro-Wilk normality test). Significance: * p≤0.05; ** p≤0.01; *** p≤0.001. ns: non-significant. RV: right ventricle. LV: left ventricle. ARG= arginine. ORN= ornithine. GUA= guanidinoacetate.
